# Hexaaqua­zinc(II) bis­(4-hydroxy­benzene­sulfonate) dihydrate

**DOI:** 10.1107/S1600536809040604

**Published:** 2009-10-10

**Authors:** Zhi-Biao Zhu, Shan Gao, Seik Weng Ng

**Affiliations:** a College of Chemistry and Materials Science, Heilongjiang University, Harbin 150080, People’s Republic of China; b Department of Chemistry, University of Malaya, 50603 Kuala Lumpur, Malaysia

## Abstract

In the crystal structure of the title compound, [Zn(H_2_O)_6_](C_6_H_5_O_4_S)_2_·2H_2_O, the Zn^II^ atom lies on a center of inversion. The complex cation inter­acts with the anion and uncoord­inated water mol­ecules by O—H⋯O hydrogen bonds, generating a three-dimensional network. The anion is disordered over two equal positions along the hydr­oxy–sulfonate C—C axis.

## Related literature

The hexa­aqua­nickel, hexa­aqua­cobalt and hexa­aqua­copper salts are not isostructural; see: Du *et al.* (2007 ▶); Kosnic *et al.* (1992 ▶); Liu & Zeng (2007 ▶).
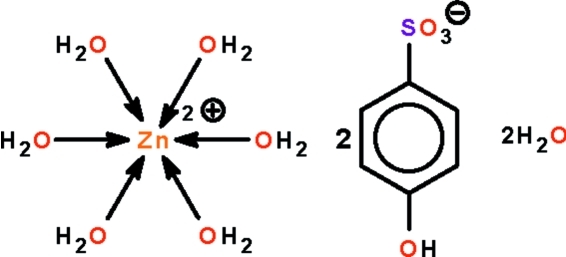


## Experimental

### 

#### Crystal data

[Zn(H_2_O)_6_](C_6_H_5_O_4_S)_2_·2H_2_O
*M* *_r_* = 555.82
Triclinic, 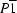

*a* = 6.2763 (5) Å
*b* = 7.0509 (7) Å
*c* = 13.3151 (11) Å
α = 78.479 (3)°
β = 76.832 (2)°
γ = 88.051 (3)°
*V* = 562.15 (9) Å^3^
*Z* = 1
Mo *K*α radiation
μ = 1.35 mm^−1^
*T* = 293 K
0.23 × 0.18 × 0.15 mm

#### Data collection

Rigaku R-AXIS RAPID IP diffractometer
Absorption correction: multi-scan (*ABSCOR*; Higashi, 1995 ▶) *T*_min_ = 0.746, *T*_max_ = 0.823
5523 measured reflections
2538 independent reflections
2283 reflections with *I* > 2σ(*I*)
*R*_int_ = 0.026

#### Refinement

*R*[*F* ^2^ > 2σ(*F* ^2^)] = 0.027
*wR*(*F* ^2^) = 0.076
*S* = 1.04
2538 reflections
214 parameters
26 restraints
H atoms treated by a mixture of independent and constrained refinement
Δρ_max_ = 0.35 e Å^−3^
Δρ_min_ = −0.47 e Å^−3^

### 

Data collection: *RAPID-AUTO* (Rigaku, 1998 ▶); cell refinement: *RAPID-AUTO*; data reduction: *CrystalClear* (Rigaku/MSC, 2002 ▶); program(s) used to solve structure: *SHELXS97* (Sheldrick, 2008 ▶); program(s) used to refine structure: *SHELXL97* (Sheldrick, 2008 ▶); molecular graphics: *X-SEED* (Barbour, 2001 ▶); software used to prepare material for publication: *publCIF* (Westrip, 2009 ▶).

## Supplementary Material

Crystal structure: contains datablocks global, I. DOI: 10.1107/S1600536809040604/xu2624sup1.cif

Structure factors: contains datablocks I. DOI: 10.1107/S1600536809040604/xu2624Isup2.hkl

Additional supplementary materials:  crystallographic information; 3D view; checkCIF report

## Figures and Tables

**Table 1:** Hydrogen-bond geometry (Å, °)

*D*—H⋯*A*	*D*—H	H⋯*A*	*D*⋯*A*	*D*—H⋯*A*
O1w—H11⋯O1	0.82 (1)	2.00 (1)	2.817 (2)	175 (3)
O1w—H12⋯O3^i^	0.83 (1)	1.97 (1)	2.801 (2)	176 (2)
O2w—H21⋯O1^ii^	0.84 (1)	1.99 (1)	2.818 (2)	168 (2)
O2w—H22⋯O2^iii^	0.84 (1)	1.91 (1)	2.730 (2)	165 (2)
O3w—H31⋯O3^iv^	0.83 (1)	2.04 (1)	2.845 (2)	166 (3)
O3w—H32⋯O4^v^	0.83 (1)	2.02 (1)	2.827 (2)	163 (3)
O4w—H41⋯O1	0.83 (1)	2.02 (1)	2.837 (2)	166 (3)
O4w—H42⋯O2^iii^	0.83 (1)	2.05 (1)	2.853 (2)	162 (3)
O4—H4⋯O4w^vi^	0.83 (1)	1.79 (1)	2.615 (2)	176 (3)

Symmetry codes: (i) 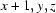; (ii) 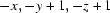; (iii) 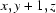; (iv) 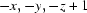; (v) 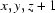; (vi) 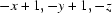.

**Fig. 1.**
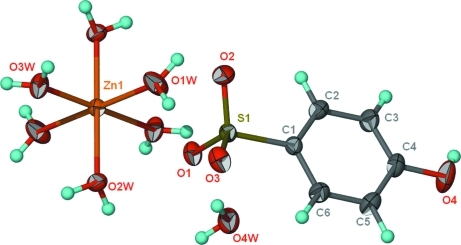
Thermal ellipsoid plot (Barbour, 2001) of [Zn(H2O)6] 2[C6H5O4S].2H2O at the 50% probability level; hydrogen atoms are drawn as spheres of arbitrary radius. The disorder in the aromatic ring is not shown.

## supplementary crystallographic information

### Experimental

Sodium 3-carboxy-4-hydroxybenzenesulfonate (0.52 g, 2 mmol) was reacted with zinc carbonate (0.25 g, 2 mmol) in water. The mixture was sealed in a 50-ml Teflon-lined stainless-steel bomb and heat at 403 K for three days. The bomb was then allowed to cool naturally to room temperature. Colorless prismatic crystals were obtained. C&H elemental analysis. Calc. for C_12_H_26_O_16_S_2_Zn: C 25.93, H 4.71%; found: C 25.97, H 4.77%. The carboxyl group of sodium 3-carboxy-4-hydroxybenzenesulfonate was apparently cleaved under the hydrothermal conditions.

### Refinement

The aromatic ring is disordered over two positions in respect of four carbon atoms. 1,2-Related carbon-carbon distances were restrained to 1.39±0.01 Å and the 1,4-related ones to 2.78±0.01 Å. Each component ring was restrained to be nearly flat. As the disorder refined to nearly 1:1, the occupancy of the disordered atoms was set as exactly 0.5.

Carbon-bound H-atoms were placed in calculated positions (C—H 0.93 Å) and were included in the refinement in the riding model approximation, with *U*(H) set to 1.2*U*(C). The water and hydroxy H-atoms were refined with a distance restraint of N–H 0.84±0.01 Å; their temperature factors were refined.

### Crystal data

**Table:**

[Zn(H_2_O)_6_](C_6_H_5_O_4_S)_2_·2H_2_O	*Z* = 1
*M**_r_* = 555.82	*F*(000) = 288
Triclinic, *P*1	*D*_x_ = 1.642 Mg m^−^^3^
Hall symbol: -P 1	Mo *K*α radiation, λ = 0.71073 Å
*a* = 6.2763 (5) Å	Cell parameters from 4800 reflections
*b* = 7.0509 (7) Å	θ = 3.1–27.5°
*c* = 13.3151 (11) Å	µ = 1.35 mm^−^^1^
α = 78.479 (3)°	*T* = 293 K
β = 76.832 (2)°	Prism, colorless
γ = 88.051 (3)°	0.23 × 0.18 × 0.15 mm
*V* = 562.15 (9) Å^3^	

### Data collection

**Table:**

Rigaku R-AXIS RAPID IP diffractometer	2538 independent reflections
Radiation source: fine-focus sealed tube	2283 reflections with *I* > 2σ(*I*)
graphite	*R*_int_ = 0.026
ω scans	θ_max_ = 27.5°, θ_min_ = 3.1°
Absorption correction: multi-scan (*ABSCOR*; Higashi, 1995)	*h* = −8→7
*T*_min_ = 0.746, *T*_max_ = 0.823	*k* = −9→9
5523 measured reflections	*l* = −17→16

### Refinement

**Table:**

Refinement on *F*^2^	Primary atom site location: structure-invariant direct methods
Least-squares matrix: full	Secondary atom site location: difference Fourier map
*R*[*F*^2^ > 2σ(*F*^2^)] = 0.027	Hydrogen site location: inferred from neighbouring sites
*wR*(*F*^2^) = 0.076	H atoms treated by a mixture of independent and constrained refinement
*S* = 1.04	*w* = 1/[σ^2^(*F*_o_^2^) + (0.0503*P*)^2^ + 0.0371*P*] where *P* = (*F*_o_^2^ + 2*F*_c_^2^)/3
2538 reflections	(Δ/σ)_max_ = 0.001
214 parameters	Δρ_max_ = 0.35 e Å^−^^3^
26 restraints	Δρ_min_ = −0.47 e Å^−^^3^

### Fractional atomic coordinates and isotropic or equivalent isotropic displacement parameters (Å^2^)

**Table:**

	*x*	*y*	*z*	*U*_iso_*/*U*_eq_	Occ. (<1)
Zn1	0.5000	0.5000	0.5000	0.02554 (10)	
S1	0.02762 (6)	0.11701 (5)	0.29910 (3)	0.02660 (11)	
O1	0.0715 (2)	0.28397 (17)	0.34183 (9)	0.0345 (3)	
O2	0.1531 (2)	−0.04947 (19)	0.33502 (10)	0.0451 (3)	
O3	−0.2054 (2)	0.07467 (19)	0.31965 (10)	0.0394 (3)	
O4	0.3707 (3)	0.3376 (3)	−0.15810 (11)	0.0584 (4)	
H4	0.5063 (16)	0.337 (4)	−0.1732 (19)	0.051 (7)*	
O1W	0.4557 (2)	0.2913 (2)	0.41675 (12)	0.0468 (4)	
H11	0.349 (3)	0.290 (4)	0.3908 (19)	0.062 (7)*	
H12	0.556 (3)	0.232 (3)	0.3850 (15)	0.043 (6)*	
O2W	0.2082 (2)	0.62516 (18)	0.47666 (9)	0.0329 (3)	
H21	0.115 (3)	0.637 (3)	0.5314 (12)	0.052 (7)*	
H22	0.207 (4)	0.7338 (19)	0.4375 (14)	0.047 (6)*	
O3W	0.3176 (2)	0.32548 (19)	0.63807 (9)	0.0377 (3)	
H31	0.292 (4)	0.2107 (17)	0.639 (2)	0.061 (7)*	
H32	0.313 (4)	0.346 (4)	0.6979 (11)	0.060 (7)*	
O4W	0.2020 (3)	0.6528 (2)	0.21483 (10)	0.0459 (3)	
H41	0.142 (4)	0.551 (3)	0.2522 (18)	0.073 (9)*	
H42	0.159 (4)	0.729 (3)	0.2548 (18)	0.064 (8)*	
C1	0.1270 (3)	0.1789 (2)	0.16170 (12)	0.0269 (3)	
C4	0.2937 (3)	0.2822 (3)	−0.05256 (13)	0.0371 (4)	
C2	0.3442 (7)	0.1495 (7)	0.1195 (3)	0.0359 (9)	0.50
H2	0.4364	0.0899	0.1639	0.043*	0.50
C3	0.4286 (7)	0.2065 (7)	0.0124 (3)	0.0376 (11)	0.50
H3	0.5803	0.1929	−0.0160	0.045*	0.50
C5	0.0730 (6)	0.3207 (7)	−0.0106 (3)	0.0390 (8)	0.50
H5	−0.0173	0.3845	−0.0550	0.047*	0.50
C6	−0.0097 (6)	0.2634 (7)	0.0973 (3)	0.0349 (8)	0.50
H6	−0.1598	0.2821	0.1267	0.042*	0.50
C2'	0.3409 (6)	0.2436 (7)	0.1239 (3)	0.0324 (9)	0.50
H2'	0.4300	0.2539	0.1715	0.039*	0.50
C3'	0.4255 (8)	0.2935 (7)	0.0161 (3)	0.0374 (11)	0.50
H3'	0.5736	0.3353	−0.0103	0.045*	0.50
C5'	0.0822 (6)	0.2055 (7)	−0.0137 (3)	0.0416 (9)	0.50
H5'	−0.0043	0.1873	−0.0611	0.050*	0.50
C6'	−0.0028 (6)	0.1555 (7)	0.0946 (3)	0.0335 (7)	0.50
H6'	−0.1480	0.1061	0.1214	0.040*	0.50

### Atomic displacement parameters (Å^2^)

**Table:**

	*U*^11^	*U*^22^	*U*^33^	*U*^12^	*U*^13^	*U*^23^
Zn1	0.02646 (15)	0.02624 (15)	0.02455 (15)	0.00073 (9)	−0.00629 (10)	−0.00595 (9)
S1	0.0311 (2)	0.0229 (2)	0.02348 (19)	−0.00025 (15)	−0.00199 (16)	−0.00367 (13)
O1	0.0408 (7)	0.0331 (6)	0.0298 (6)	−0.0060 (5)	−0.0032 (5)	−0.0110 (4)
O2	0.0593 (9)	0.0357 (7)	0.0329 (6)	0.0145 (6)	−0.0056 (6)	0.0030 (5)
O3	0.0338 (6)	0.0404 (7)	0.0410 (7)	−0.0092 (5)	0.0023 (5)	−0.0117 (5)
O4	0.0538 (10)	0.0940 (13)	0.0232 (6)	−0.0074 (9)	−0.0055 (7)	−0.0044 (7)
O1W	0.0357 (7)	0.0558 (9)	0.0648 (9)	0.0112 (6)	−0.0203 (7)	−0.0403 (7)
O2W	0.0306 (6)	0.0357 (7)	0.0282 (6)	0.0056 (5)	−0.0048 (5)	0.0005 (5)
O3W	0.0504 (8)	0.0336 (7)	0.0254 (6)	−0.0108 (6)	−0.0037 (5)	−0.0009 (5)
O4W	0.0596 (9)	0.0382 (8)	0.0342 (7)	−0.0030 (6)	−0.0011 (6)	−0.0044 (6)
C1	0.0320 (8)	0.0238 (7)	0.0236 (7)	0.0008 (6)	−0.0041 (6)	−0.0044 (5)
C4	0.0449 (10)	0.0421 (10)	0.0240 (8)	−0.0014 (8)	−0.0075 (7)	−0.0061 (7)
C2	0.039 (2)	0.038 (2)	0.0278 (18)	0.007 (2)	−0.0079 (15)	−0.0010 (18)
C3	0.033 (2)	0.047 (3)	0.0263 (19)	0.002 (2)	0.0006 (15)	−0.002 (2)
C5	0.044 (2)	0.043 (2)	0.0318 (19)	−0.0016 (18)	−0.0157 (16)	−0.0018 (16)
C6	0.0322 (19)	0.040 (2)	0.0327 (18)	0.0040 (17)	−0.0096 (14)	−0.0059 (16)
C2'	0.0311 (18)	0.045 (2)	0.0228 (16)	−0.0036 (19)	−0.0072 (13)	−0.0081 (18)
C3'	0.034 (2)	0.043 (3)	0.032 (2)	−0.003 (2)	0.0011 (15)	−0.008 (2)
C5'	0.046 (2)	0.055 (3)	0.0289 (18)	0.001 (2)	−0.0176 (16)	−0.0102 (18)
C6'	0.0317 (18)	0.039 (2)	0.0320 (18)	−0.0016 (16)	−0.0082 (14)	−0.0093 (16)

### Geometric parameters (Å, °)

**Table:**

Zn1—O2W^i^	2.0660 (11)	C1—C6'	1.375 (4)
Zn1—O2W	2.0660 (11)	C1—C2	1.377 (4)
Zn1—O1W	2.0713 (13)	C1—C2'	1.380 (4)
Zn1—O1W^i^	2.0713 (13)	C1—C6	1.387 (4)
Zn1—O3W	2.1066 (12)	C4—C3	1.367 (4)
Zn1—O3W^i^	2.1066 (12)	C4—C3'	1.380 (5)
S1—O2	1.4530 (12)	C4—C5'	1.395 (4)
S1—O3	1.4552 (13)	C4—C5	1.409 (4)
S1—O1	1.4651 (12)	C2—C3	1.384 (5)
S1—C1	1.7624 (15)	C2—H2	0.9500
O4—C4	1.358 (2)	C3—H3	0.9500
O4—H4	0.83 (1)	C5—C6	1.392 (5)
O1W—H11	0.82 (1)	C5—H5	0.9500
O1W—H12	0.83 (1)	C6—H6	0.9500
O2W—H21	0.84 (1)	C2'—C3'	1.389 (5)
O2W—H22	0.84 (1)	C2'—H2'	0.9500
O3W—H31	0.83 (1)	C3'—H3'	0.9500
O3W—H32	0.83 (1)	C5'—C6'	1.395 (5)
O4W—H41	0.83 (1)	C5'—H5'	0.9500
O4W—H42	0.83 (1)	C6'—H6'	0.9500
			
O2W^i^—Zn1—O2W	180.000 (1)	C6'—C1—S1	120.64 (18)
O2W^i^—Zn1—O1W	90.22 (5)	C2—C1—S1	119.18 (18)
O2W—Zn1—O1W	89.78 (5)	C2'—C1—S1	118.08 (17)
O2W^i^—Zn1—O1W^i^	89.78 (5)	C6—C1—S1	120.43 (18)
O2W—Zn1—O1W^i^	90.22 (5)	O4—C4—C3	121.5 (2)
O1W—Zn1—O1W^i^	180.0	O4—C4—C3'	120.7 (2)
O2W^i^—Zn1—O3W	92.58 (5)	O4—C4—C5'	119.52 (19)
O2W—Zn1—O3W	87.42 (5)	C3—C4—C5'	111.2 (3)
O1W—Zn1—O3W	89.15 (6)	C3'—C4—C5'	119.8 (3)
O1W^i^—Zn1—O3W	90.85 (6)	O4—C4—C5	117.71 (19)
O2W^i^—Zn1—O3W^i^	87.42 (5)	C3—C4—C5	120.6 (3)
O2W—Zn1—O3W^i^	92.58 (5)	C3'—C4—C5	112.2 (3)
O1W—Zn1—O3W^i^	90.85 (6)	C1—C2—C3	120.2 (3)
O1W^i^—Zn1—O3W^i^	89.15 (6)	C1—C2—H2	119.9
O3W—Zn1—O3W^i^	180.0	C3—C2—H2	119.9
O2—S1—O3	112.90 (9)	C4—C3—C2	120.0 (4)
O2—S1—O1	110.94 (8)	C4—C3—H3	120.0
O3—S1—O1	112.10 (7)	C2—C3—H3	120.0
O2—S1—C1	105.84 (7)	C6—C5—C4	118.6 (3)
O3—S1—C1	107.93 (8)	C6—C5—H5	120.7
O1—S1—C1	106.68 (7)	C4—C5—H5	120.7
C4—O4—H4	110.5 (17)	C1—C6—C5	120.0 (3)
Zn1—O1W—H11	122.4 (17)	C1—C6—H6	120.0
Zn1—O1W—H12	124.5 (16)	C5—C6—H6	120.0
H11—O1W—H12	109 (2)	C1—C2'—C3'	119.9 (3)
Zn1—O2W—H21	115.9 (16)	C1—C2'—H2'	120.1
Zn1—O2W—H22	120.5 (16)	C3'—C2'—H2'	120.1
H21—O2W—H22	103 (2)	C4—C3'—C2'	119.7 (4)
Zn1—O3W—H31	119.8 (18)	C4—C3'—H3'	120.1
Zn1—O3W—H32	122.9 (19)	C2'—C3'—H3'	120.1
H31—O3W—H32	112 (2)	C4—C5'—C6'	120.3 (3)
H41—O4W—H42	100 (3)	C4—C5'—H5'	119.9
C6'—C1—C2	111.5 (2)	C6'—C5'—H5'	119.9
C6'—C1—C2'	121.2 (2)	C1—C6'—C5'	118.9 (3)
C2—C1—C6	120.3 (2)	C1—C6'—H6'	120.6
C2'—C1—C6	112.6 (2)	C5'—C6'—H6'	120.6
			
O2—S1—C1—C6'	112.2 (3)	C5'—C4—C5—C6	76.9 (5)
O3—S1—C1—C6'	−8.9 (3)	C6'—C1—C6—C5	−83.1 (5)
O1—S1—C1—C6'	−129.6 (2)	C2—C1—C6—C5	−0.7 (6)
O2—S1—C1—C2	−32.7 (3)	C2'—C1—C6—C5	30.0 (5)
O3—S1—C1—C2	−153.8 (3)	S1—C1—C6—C5	176.7 (3)
O1—S1—C1—C2	85.5 (3)	C4—C5—C6—C1	3.1 (6)
O2—S1—C1—C2'	−65.2 (3)	C6'—C1—C2'—C3'	2.6 (6)
O3—S1—C1—C2'	173.7 (2)	C2—C1—C2'—C3'	79.8 (6)
O1—S1—C1—C2'	53.1 (3)	C6—C1—C2'—C3'	−32.5 (5)
O2—S1—C1—C6	149.9 (2)	S1—C1—C2'—C3'	179.9 (4)
O3—S1—C1—C6	28.7 (3)	O4—C4—C3'—C2'	177.4 (4)
O1—S1—C1—C6	−91.9 (3)	C3—C4—C3'—C2'	−82.7 (8)
C6'—C1—C2—C3	35.6 (5)	C5'—C4—C3'—C2'	−5.3 (6)
C2'—C1—C2—C3	−80.8 (6)	C5—C4—C3'—C2'	31.8 (5)
C6—C1—C2—C3	0.9 (6)	C1—C2'—C3'—C4	1.4 (7)
S1—C1—C2—C3	−176.6 (4)	O4—C4—C5'—C6'	−177.3 (4)
O4—C4—C3—C2	−179.3 (4)	C3—C4—C5'—C6'	33.0 (5)
C3'—C4—C3—C2	84.4 (8)	C3'—C4—C5'—C6'	5.4 (6)
C5'—C4—C3—C2	−30.3 (6)	C5—C4—C5'—C6'	−80.6 (5)
C5—C4—C3—C2	6.3 (6)	C2—C1—C6'—C5'	−32.4 (5)
C1—C2—C3—C4	−3.7 (7)	C2'—C1—C6'—C5'	−2.5 (6)
O4—C4—C5—C6	179.4 (4)	C6—C1—C6'—C5'	80.8 (5)
C3—C4—C5—C6	−6.0 (6)	S1—C1—C6'—C5'	−179.8 (3)
C3'—C4—C5—C6	−33.9 (5)	C4—C5'—C6'—C1	−1.5 (6)

Symmetry codes: (i) −*x*+1, −*y*+1, −*z*+1.

### Hydrogen-bond geometry (Å, °)

**Table:**

*D*—H···*A*	*D*—H	H···*A*	*D*···*A*	*D*—H···*A*
O1w—H11···O1	0.82 (1)	2.00 (1)	2.817 (2)	175 (3)
O1w—H12···O3^ii^	0.83 (1)	1.97 (1)	2.801 (2)	176 (2)
O2w—H21···O1^iii^	0.84 (1)	1.99 (1)	2.818 (2)	168 (2)
O2w—H22···O2^iv^	0.84 (1)	1.91 (1)	2.730 (2)	165 (2)
O3w—H31···O3^v^	0.83 (1)	2.04 (1)	2.845 (2)	166 (3)
O3w—H32···O4^vi^	0.83 (1)	2.02 (1)	2.827 (2)	163 (3)
O4w—H41···O1	0.83 (1)	2.02 (1)	2.837 (2)	166 (3)
O4w—H42···O2^iv^	0.83 (1)	2.05 (1)	2.853 (2)	162 (3)
O4—H4···O4w^vii^	0.83 (1)	1.79 (1)	2.615 (2)	176 (3)

Symmetry codes: (ii) *x*+1, *y*, *z*; (iii) −*x*, −*y*+1, −*z*+1; (iv) *x*, *y*+1, *z*; (v) −*x*, −*y*, −*z*+1; (vi) *x*, *y*, *z*+1; (vii) −*x*+1, −*y*+1, −*z*.
